# The Effect of Culture Medium Obtained from Dental Pulp Stem Cells (Conditioned Medium) on Esophageal Squamous Cell Carcinoma Cell Line (KYSE-30)

**DOI:** 10.22038/ijorl.2025.84920.3857

**Published:** 2026

**Authors:** Setare Tavakoli, Akbar Safipour Afshar, Nooshin Mohtasham, Zohre Samiee, Mohammad Amin Yaghoubi, Farnaz Mohajertehran

**Affiliations:** 1 *Department of Biology, Neyshabur Branch, Islamic Azad University, Neyshabur, Iran. *; 2 *Department of Oral and Maxillofacial Pathology, School of Dentistry, Mashhad University of Medical Sciences, Mashhad, Iran.*; 3 *Food Control Laboratory, Department of Food and Drug, Mashhad University of Medical Sciences, Mashhad, Iran.*; 4 *Dental Research Center, Mashhad University of Medical Sciences, Mashhad, Iran.*

## Abstract

**Introduction:**

Esophageal squamous cell carcinoma (ESCC) is the eighth most prevalent cancer worldwide. Head and neck squamous cell carcinoma (HNSCC) represents over 90% of all head and neck cancers, and nearly 40% of patients fail treatment. Therefore, discussion of cancer treatment is essential. In this in vitro study, we examined how conditioned medium from dental pulp stem cells (DPSC-CM) influences the ESCC cell line (KYSE-30).

**Materials and Methods:**

First, the middle pulp tissue of the wisdom tooth was extracted and, following sterilization, transferred to a cell culture flask containing MEM-α. After completing the culture procedures, conditioned medium (CM) was collected from the fourth passage culture after 72 hours in serum-free medium. The KYSE-30 cells were then treated with the CM for an additional 72 hours, and cell survival was assessed using the MTT assay. Statistical significance was evaluated using one-way ANOVA followed by Tukey’s post-hoc test.

**Results:**

The results demonstrated that treatment of esophageal cancer cells with conditioned medium (CM) significantly reduced the survival rate of cancer cells compared to control samples. The treatment of KYSE-30 cells with DPSC-CM led to a notable reduction in cell viability (CM group: 51.2 ± 4.1% vs. control group: 72.6 ± 3.3%; P = 0.004).

**Conclusion:**

DPSC-CM demonstrates the ability to reduce the proliferation of cancer cells; therefore, this medium can be considered a potential drug (therapeutic) candidate for the treatment of esophageal cancer. However, further studies are required to confirm these results.

## Introduction

Esophageal cancer ranks as one of the most prevalent types of cancer in the world, with an even higher incidence in Iran. It occurs when a malignant tumor develops in the wall of the esophagus. This tumor’s growth can influence the deeper muscular and connective tissues of the esophagus. The tumor can appear anywhere along the length of the esophagus (1). Esophageal cancer ranks 8th in prevalence and 6th in mortality among cancers worldwide. Some risk factors associated with this cancer include race, gender, obesity, Helicobacter pylori bacteria, human papillomavirus, and consumption of tobacco and alcohol (2-4). This cancer can affect various tissues of the esophagus, resulting in different types of cancer. SCC and adenocarcinoma are the two most common histological forms of esophageal cancer. SCC is the most prevalent type of esophageal cancer worldwide, and its incidence is significantly influenced by geographic region. Typically, these cancers develop in the cervical and thoracic segments of the esophagus (5-7).

Esophageal carcinoma has a poor prognosis, with only 10% of patients surviving five years after diagnosis. Therefore, the goal of treatment is to control the symptoms. There are various treatment methods, including surgery, chemotherapy, radiation therapy, and the utilization of stem cell therapy (8-12).

**Fig 1:**
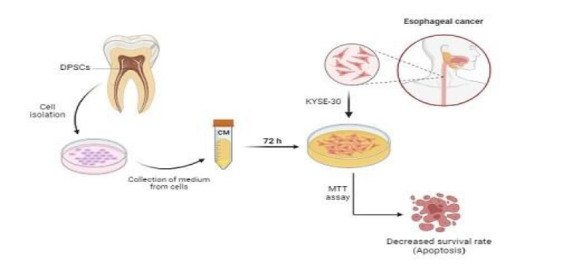
**.** Influence of Dental Pulp Stem Cell-Derived Conditioned Medium on the KYSE-30 Esophageal Squamous Cell Carcinoma Line

There are two significant categories of stem cells: embryonic stem cells and adult stem cells. Among the adult stem cells, mesenchymal stem cells (MSCs) have received a lot of attention from researchers. These cells are highly proliferative, multipotent, and capable of differentiating into various types of mesenchymal cells (13-15). The tooth contains various stem cells derived from both mesenchymal and epithelial origins. The stem cells derived from the dental pulp are called dental pulp stem cells (DPSCs). These cells are mostly isolated from wisdom teeth and have the same characteristics as MSCs derived from bone marrow (BM-MSC) (16).DPSCs can express markers characteristic of MSCs, such as STRO-1. These cells are multipotent, capable of differentiating into various cell types, including odontoblasts, adipocytes, chondrocytes, and osteoblasts(17,18). The easy isolation of dental mesenchymal stem cells through less invasive approaches has made them promising alternatives to non-dental MSCs. It has been observed that cytokines such as VEGF, BFGF, EGF, TGF-β, HGF, PDGF, KGF, TNF, and IL-6 are present in the conditioned medium (CM) obtained from MSCs under hypoxic circumstances. These factors, known as microvesicles, secretome, or exosomes, have been identified within the culture medium of stem cells, known as CM. Because there are no cells, graft rejection and immunological reactions do not occur when employing CM. Among the other benefits of the CM are its antioxidant and antitumor effects. On the other hand, unlike stem cells, CM does not pose the risk of cancer (Figure 1) (19,20). 

 Recent experimental studies have highlighted the potential anti-cancer properties of dental pulp stem cell-conditioned medium (DPSC-CM) in multiple cancer models. For instance, Nikkhah et al. (2021) demonstrated that DPSC-CM significantly reduced colorectal cancer cell viability and migration, while promoting apoptosis via the MAPKinase and caspase pathways (20,21). Similarly, Raj et al. (2021) reported that DPSC-CM decreased Ki‑67 expression in oral SCC cells, although the reduction was not statistically significant (21). More recently, an in vitro study on head and neck SCC cell lines showed that DPSC secretome enhanced apoptosis, reduced cell proliferation, and sensitized cells to chemotherapy agents like Taxotere (22). Despite these promising findings in colorectal, oral, and head & neck cancers, little is known about how DPSCCM influences esophageal SCC (23). Our study is among the first to investigate the therapeutic potential of DPSC‑CM on the KYSE‑30 ESCC cell line at the molecular and functional level.

## Materials and Methods

### KYSE-30 culture

**Fig 2:**
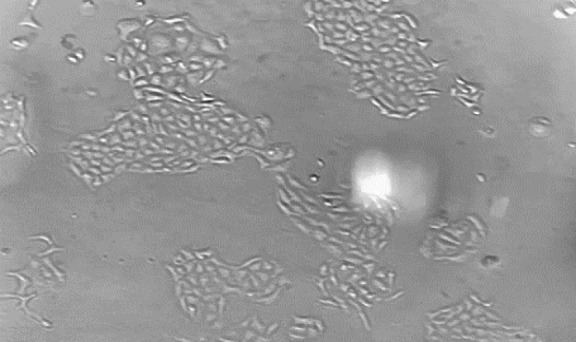
Morphological changes in KYSE-30 cells before and after 72-hour treatment with DPSC-CM. (A) Control cells with normal morphology. (B) Treated cells showing reduced density and predo- minantly small, dead cells. (10X magnification).

This in vitro investigation was performed according to the appropriate guidelines provided by the Organizational Ethics Committee of Mashhad University of Medical Sciences, Iran (IR. MUMS. DENTISTRY. REC.1400.078) from September 2022 to April 2024. KYSE-30 cell line (Human Epithelial Esophageal SCC) was purchased from the Pasteur Institute of Iran cell bank. The cells were cultured in DMEM High Glucose medium with 10% FBS and 1% antibiotic (penicillin-streptomycin) (P/S), maintained at 37°C in an atmosphere of 5% CO₂ and 95% humidity (Figure 2).

### Isolation of DPSCs

 In order to obtain DPSCs, first, a perfectly healthy wisdom tooth was removed from the tissue and promptly rinsed with physiological serum following extraction. It was then cleansed with betadine solution and 70% alcohol before being transported to the Faculty of Dentistry of Mashhad University of Medical Sciences in a sterile Falcon tube filled with PBS. Under sterile conditions, the dental pulp was extracted using forceps and transferred to a sterile microtube containing PBS, 0.5 cc of trypsin, and 1% penicillin, then rinsed at least 6 times. It was then placed in a sterile plate and divided into 1mm pieces. Afterwards, the pieces were transferred to a microtube containing 1cc of type I collagenase and dispase and incubated for one hour at 37°C. In another method, the pieces were placed in a microtube containing 1cc of trypsin and incubated at 37°C. Then the pieces were moved to a 6-well plate and placed in the vicinity of MEM, a culture medium to neutralize the enzyme activity. The culture medium was substituted after 10 days. Following the cell passage, the supernatant medium was collected and centrifuged at 2000 RPM for five minutes, then stored at -70°C for future use.

### Identifying DPSCs by surface markers using flow cytometry

The identity of the isolated cells was confirmed using flow cytometry by measuring the expression of surface markers CD45, CD29, CD44, and CD34. In this method, the cells were trypsinized in the third passage and washed with FBS following cell counting. They were then incubated for 30 minutes at 4°C with Mouse Anti-Human CD34 Fluorescein isothiocyanate (FITC), Mouse Anti-Human CD45 FITC, Mouse Anti-Human CD44 FITC, and Mouse Anti-Human CD29 FITC. They were ultimately washed with a PBS solution to remove excess antibodies. The results were analyzed with the BECTON DICKINSON flow cytometer.

### Collection of CM

After performing the culture steps, CM was collected from the fourth pulp passage in a serum-free medium after 72 hours. In the next step, KYSE-30 was exposed to CM for 72 hours, and the proliferation and survival rates of the cells were determined by MTT and cell counting (hemocytometry).

### Assessing cell survival by MTT

After preparing the KYSE-30 cancer cells, a total of 2.1 × 10⁶ cells were seeded into a 6-well plate (1.2 mL per well). After 24 hours, the following treatment groups were established:

- Group 1 (Control): Cells received only complete culture medium (DMEM + 10% FBS).
- Group 2: Cells received a 1:1 mixture of DPSC-CM and complete culture medium.
- Group 3 (CM only): Cells received 100% DPSC-conditioned medium (serum-free).

All groups were incubated for 72 hours. Then, cell viability was assessed via the MTT assay, with 100 µL of medium and 10 µL of MTT solution added to each well, and plates were incubated at 37°C for 4 hours. Afterward, 85 µL of supernatant was discarded and 50 µL of DMSO was added. The absorbance was read at 600 nm using an ELISA plate reader. All treatments were performed in triplicate.

### Data analysis

Experiments were repeated three times to ensure reliable and consistent results. Data are expressed as mean ± SD of three separate replicates. Statistical analysis was conducted using one-way analysis of variance (ANOVA) to assess overall differences between treatment groups. When the ANOVA results were significant, Tukey’s post-hoc test was applied for pairwise comparisons between groups. A p-value < 0.05 was considered statistically significant. Data analysis was performed using SPSS version 22.0.

## Results

### Flow cytometry results

**Fig 3:**
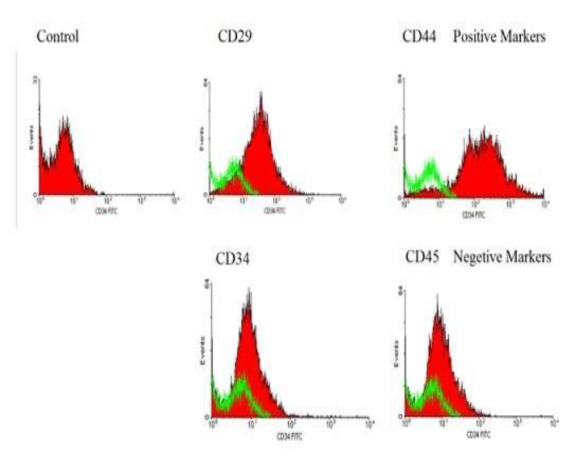
Flow cytometry histograms showing the expression of mesenchymal (CD29, CD44) and hematopoietic (CD34, CD45) surface markers in DPSC populations. Red histograms indicate antibody-stained cells; green histograms represent isotype controls.

Figure 3 illustrates the display of surface markers on DPSCs. The results indicated that the expression of CD34 (primary hematopoietic progenitor cell and endothelial cell marker) and CD45 (leukocyte progenitor stem cell marker) was negative, and the expression of CD29 and CD44 (mesenchymal stem cell markers) was highly positive. In other words, compared with the control group, CD29 and CD44 showed a right shift, while the negative markers CD45 and CD34 peaked in the same range as the control group. Isotype-matched FITC-conjugated antibodies were used as negative controls to set the gating strategy for CD34, CD45, CD44, and CD29 markers.

### MTT Assay

Cell survival was assessed using the MTT assay after 72 hours of incubation. The absorption rate of cells with CM was lower compared to the cells containing either culture medium or both culture medium and CM. Results from the MTT assay indicated that treatment with DPSC-CM significantly decreased the viability of KYSE-30 cells relative to the control group.

**Fig 4:**
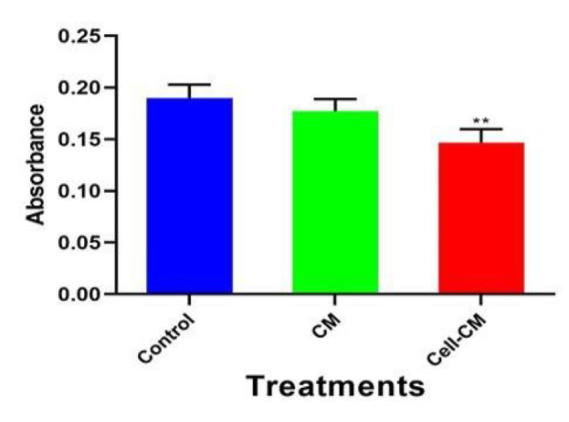
Bar graph showing the viability of KYSE-30 cells after 72 hours of treatment with different media: control (culture medium only), 50% CM + 50% medium, and 100% CM. Cell viability was measured using MTT assay. Values represent mean ± SD (n = 3). P < 0.05 compared to control.

The mean cell viability was: Control group: 72.6 ± 3.3%, 50% CM group: 61.4 ± 2.7% , 100% CM group: 51.2 ± 4.1% . The groups exhibited statistically significant differences (P = 0.004), as determined by one-way ANOVA. (Table 1, Figure 4)

**Table 1:** Viability (%) of KYSE-30 cells after 72 hours of treatment with DPSC-CM

** Group**	**Mean** ** viability (%)**	**Standard ** **deviation (SD)**	**ANOVA ** **p-value**	**Tukey post-hoc P-value vs. Control**
Control (medium only)	72.6	3.3	0.004	–
DPSC-CM 50% + medium 50%	61.4	2.7	0.004	0.018*
DPSC-CM 100%	51.2	4.1	0.004	0.004*

Statistical significance assessed using One-way ANOVA. A P-value < 0.05 was considered significant Data are expressed as mean ± SD of three independent replicates. Overall group differences were first assessed using one-way ANOVA (p = 0.004). Pairwise comparisons with the control group were then analyzed using Tukey’s post-hoc test. A p-value < 0.05 was considered statistically significant.

## Discussion

The current study revealed that DPSC-CM reduces the growth and survival of human esophageal cancer cell lines. Our findings indicate that DPSCs may be a superior source for developing novel methods in esophageal cancer treatment. It can also be employed in cancer treatment due to its toxicity on KYSE-30 cell lines. DPSCs are MSCs that have the capacity to differentiate into different cell types and exhibit self-renewal (24). They have been shown to provide benefits for both dental and systemic diseases. Comprehensive research suggests that DPSCs are effective in treating a range of conditions, including spinal cord injuries, Parkinson's disease, Alzheimer's disease, cerebral ischemia, muscular dystrophy, diabetes, eye diseases, and oral and dental diseases (25). DPSCs have also been reported to have a higher regenerative potential than BM-MSCs (26), which are regarded as typical MSCs. DPSCs have recently received much interest. Their clinical application in the jaw and facial region is currently the topic of substantial research.

For the first time, Salehi et al. employed DPSCs as carriers of anticancer drugs. The results demonstrated that DPSCs can be loaded with anticancer drugs in vitro without affecting their viability. Their results indicated that the application of DPSCs for targeted cancer treatment could be a groundbreaking approach to minimize chemotherapy-related complications and enhance the efficacy of systemic cancer treatment (27).

Cell-free derivatives are less likely to lose their functional properties. Since the secretome of DPMSCs is the primary component of DPMSCs, which can impact the rate of cell proliferation - either positively or negatively, it is advisable to employ them with caution (28). A significant advantage of conditioned medium (CM) derived from DPSC is its ability to modulate the host immune response and reduce the risk of substantial adverse effects. Cell-free secretome-based treatment of DPSC-CM offers multiple benefits over cell-based therapy, including relatively easy processing and long-term storage capabilities (29). 

In 2021, Raj et al. conducted a study to measure the levels of growth factors and cytokines in DPMSCs and to assess their impact on the proliferation of oral cancer cells (AW123516). Their results indicated that concentrations of 50% and 100% DPMSC-CM inhibited the expression of the tumor marker Ki-67 in AW13516, although this effect was not significant. This study’s findings are an example of the therapeutic properties of DPMSC-CM on oral cancers, which are consistent with our results (21). In 2021, Sarra et al. investigated the impact of DPSC-CM stem cells on the regeneration of damaged pulp tissue. The findings affirmed that DPSC-CM can aid in the restoration of the dentin-pulp complex, making it a feasible option for restorative dentistry. Although the goals of their study differ from ours, the results confirm the therapeutic properties of DPMSC-CM (30).

 Despite the exponential rise of stem cell research, very few molecular studies on the influence of oral pulp stem cells on malignancies, notably esophageal cancer, have been undertaken. Therefore, the exact mechanism of the effect of DPMSC and DPSC-CM in carcinogenicity or cancer inhibition remains unknown. Many studies demonstrate that several growth factors, including VEGF, HCF, Ang-2, TGF-α, EPO, SCF, FGF, and PDGF-BB, and pro-inflammatory cytokines TNF-α and CXCL8, can play a significant role in cancer cell proliferation (21)The role of VEGF, EGF, IL-8, and FGF-2 in angiogenesis to supply oxygen and nutrients in the microenvironment of growing tumors or metastasis has been determined (31). Additionally, the results demonstrate that the profiles of growth factors and cytokines in DPMSCs can be affected by age (32).

Studies indicate that DPMSC-CM can help cancer treatment by increasing apoptosis and inhibiting cell proliferation (33). This effect is believed to result from a reduction in the expression of the anti-apoptotic marker Bcl-2 and an upregulation of the pro-apoptotic marker BAX (34). DPMSC-CM also downregulates Col 1, fibronectin, and laminin 2, indicating a reduced ability to migrate (35). 

Although apoptotic biomarkers were not assessed in our study, the downregulation of Bcl-2 and the increase of BAX (36) Might explain the results of our research, which indicates that DPMSC-CM caused the death of esophageal cancer cells by increasing apoptotic markers.

## Conclusion

The results of this research demonstrated that treating squamous esophageal cancer cells with CM significantly reduced cancer cell survival compared to the control groups. DPSC-CM can inhibit the growth of cancer cells, indicating its potential as a drug candidate for the treatment of esophageal cancer. However, further investigation is required to validate these findings.

## Data Availability

The data underlying the findings of this study are available from Farnaz Mohajertehran; however, they are not publicly accessible due to licensing restrictions associated with this study. Data can be provided upon reasonable request, subject to approval from Farnaz Mohajertehran.
